# Role of the IGF-1 Axis in Overcoming Resistance in Breast Cancer

**DOI:** 10.3389/fcell.2021.641449

**Published:** 2021-03-22

**Authors:** Anna Ianza, Marianna Sirico, Ottavia Bernocchi, Daniele Generali

**Affiliations:** ^1^Department of Medical, Surgery and Health Sciences, Azienda Sanitaria Universitaria Giuliano Isontina, Trieste, Italy; ^2^Department of Surgery and Cancer, Faculty of Medicine, Imperial College London, London, United Kingdom; ^3^Breast Cancer Unit and Translational Research Unit, ASST Cremona, Cremona, Italy; ^4^Department of Medicine, Surgery and Health Sciences, University of Trieste, Trieste, Italy

**Keywords:** IGF1, IGF-1R, clinical trial, therapy resistance, breast cancer

## Abstract

Over the last two decades, many studies have demonstrated that the insulin-like growth factor-1 (IGF-1) is involved in a number of patho-physiological processes, as well as in the development of different types of solid tumors, including breast cancer (BC). Preclinical and clinical data showed that IGF-1 receptor (R) is overexpressed and hyper-phosphorylated in several subtypes of BCs. The central implications of this pathway in tumor cell proliferation and metastasis make it an important therapeutic target. Moreover, the IGF-1 axis has shown strong interconnection with estrogen regulation and endocrine therapy, suggesting a possible solution to anti-estrogen resistance. IGF-1R might also interfere with other pivotal therapeutic strategies, such as anti HER2 treatments and mTOR inhibitors; several clinical trials are ongoing evaluating the role of IGF-1R inhibition in modulating resistance mechanisms to target therapies. Our aim is to offer an overview of the most recent and significant field of application of IGF-1 inhibitors and relevant therapeutic strategies, weighing their possible future impact on clinical practice.

## Introduction

**FIGURE 1:**
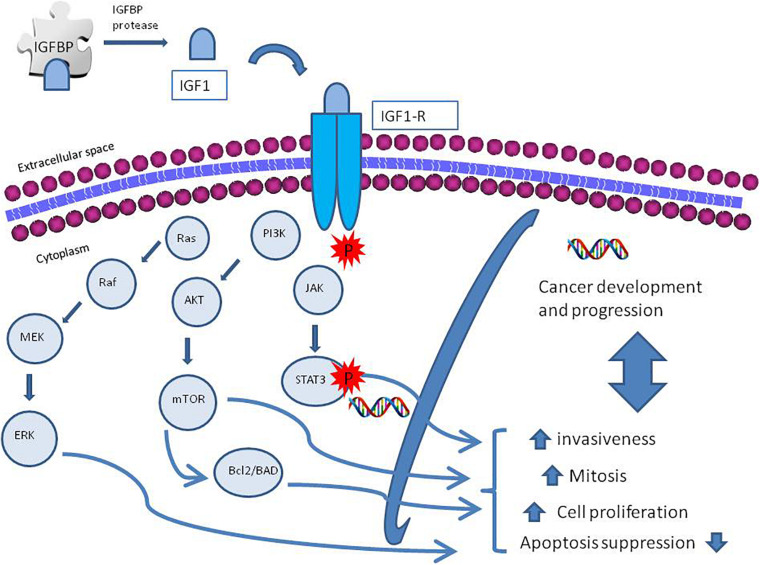
A schematic diagram of insulin growth factor-1 receptor (IGF-IR) activation and regulation. The IGF axis consists of ligands as insulin, insulin-like growth factor 1 and 2 (IGF-1, IGF-2), receptor, IGF binding proteins (IGFBPs) 1–7, and IGFBP proteases. The IGF ligands bind their receptors and binding proteins with high affinity. IGFBPs bind tightly to IGF ligands, influencing binding to their receptors; IGFBP proteases cleave the IGFBPs into fragments with lower affinity for the IGF ligands, thereby increasing free IGF-1 and IGF-2 bioavailability. Activation of IGF-1R promotes cellular growth, proliferation, survival, and metastasis via activation of molecular pathways downstream; among them the phosphatidylinositol 3-kinase (PI3K)-AKT and RAS-extracellular signal-regulated kinase (ERK) pathways.

The insulin-like growth factor-1 (IGF-1) is an insulin-like protein with anabolic effects, whose production is stimulated by growth hormone (GH), and is one of the main mediators of GH effects. Its circulating levels vary during childhood and reach its highest levels during puberty (Grimberg et al., 2016). The insulin-like growth factors (IGF-1 and IGF-2), their receptors, and a system of six insulin-growth factor binding proteins (IGFBP-1 to IGFBP-6) form a network involved in the activation of many downstream pathways (Allard and Duan, 2018). Multiple factors might activate IGF-1 receptor (R) tyrosine kinase activity (Llak, 2008) leading to interaction with its substrate, as insulin receptor (IR) substrate and the Drc-homology-2 containing protein SH2 (Radhakrishnan et al., 2011). After phosphorylation, this protein, acting as docking molecules, activates cellular kinases and initiates different downstream signaling pathways. Specifically, IGF-IR activates the PI3K/AKT/mTOR and ras/raf/MEK signaling pathways that promote cell proliferation and, at the same time, inhibits programmed cell death, through the activation of the B-cell lymphoma 2 (Bcl2)/Bcl2 antagonist of cell death (BAD) pathway, leading to carcinogenesis (Hakuno and Takahashi, 2018). The transcription of IGF-1 enables the activation of the STAT3 pathway, which enhances the invasive ability of tumor cells in prostate cancer (Ma et al., 2020); Wang et al. (2020) demonstrated that IGF-1 activates NFkB signaling inflammation via cytosolic ROS in various cell cultures. An overview of the signaling pathways is described in Figure 1.

Insulin-like growth factor-1 and its system of binding proteins and receptors are physiologically involved in the development of many human tissues (Slepicka et al., 2020). It has been suggested that IGF-1 plays a significant role in the ductal and mammary gland formation, function, and maintenance (Christopoulos et al., 2015). Preclinical and clinical data have shown that IGF-IR is overexpressed and hyper-phosphorylated in several subtypes of breast cancers (BCs) (Law et al., 2008), from which its role in BC development has stemmed. High plasma levels of IGF-1 and IGFBP-3 represent a risk factor for the development and recurrence of BC in the general population (Key et al., 2010). This is particularly verified for the incurrence of estrogen receptor positive (ER+) tumors, independent from menopausal status (Key et al., 2010). Whether it constitutes an additional risk for women with a family history of disease is not yet clarified (Monson et al., 2020). However, an Italian study associated an increased risk of BC in patients with BRCA mutation (hereditary BC) with high serum IGF-1 levels (Pasanisi et al., 2011). Moreover, its role and level regulation naturally reveal a strong connection with dysmetabolism and body mass index (BMI), especially being a risk factor in HER2 positive (HER2+) overweight patients (Tong et al., 2020). Furthermore, it has been recently suggested that this factor could hold a negative prognostic significance in BC (Hartog et al., 2013), overall and in patients undergoing endocrine therapy (Duggan et al., 2013; Hartog et al., 2013). Our aim is to offer a focused review of the possible clinical role of IGF-1 as a therapeutic target and/or as part of combination therapy in BC.

## Plasma Levels of IGF-1 and Breast Cancer

The concentrations of IG1 in plasma are approximately 150–400 ng/mL, where it is present mostly as protein-bound form (Clemmons, 2007b). The free ligand concentration is less than 1% (Clemmons, 2007b). A family of high affinity IGF binding proteins (IGFBPs) has the role of protecting IGFs from degradation through the formation of the complex IGFBP-IGF (Firth and Baxter, 2002). Even if IGFBPs were originally described as passive circulating transport proteins for IGF-I and IGF-II, now they are recognized as playing an important role in BC and IGF-1 action (Firth and Baxter, 2002). The major IGF transport function might be attributed to IGFBP-3, which is the most abounding IGF binding protein in the blood stream, followed by IGFBP-2 (Firth and Baxter, 2002). Once removed from the circulation, the binary complexes of IGFBP-IGF cross the endothelium to reach the target tissue and to interact with cell surface receptors. As the IGFBPs have a higher affinity for the IGFs than the receptors, they could sequestrate IGFs away from the type I IGF receptor, blocking their interaction. On the other hand, IGFBPs may increase IGF cellular functions in the local microenvironment by acting as a reservoir that could slowly unbind the ligands (Brahmkhatri et al., 2015).

Many different factors affect IGF-1 plasma concentrations: GH activity, nutritional status, sex, estrogen levels, and age (Clemmons and Van Wyk, 1984). Circulating IGF-1 is one of the major risk factors associated with increased BC risk (Lann and LeRoith, 2008). Previous *in vitro* studies demonstrated that IGF-1 stimulates the growth of human BC cell lines (Sasi et al., 2014; de Groot et al., 2020) and the *in vitro* blocking of IGF-1 system inhibits the response of human BC cell lines (Zha and Lackner, 2010). In the 1980s, the initial report by Furlanetto and DiCarlo (1984) highlighted the possible role of IGF-1 in the development of BC.

Later, many epidemiological and prospective studies have reported a positive correlation between circulating IGF-1 levels and BC development. A case-controlled study reported higher IGF-1 plasma concentrations in women with BC than patients without (Bruchim et al., 2009). Additionally, Werner and Laron (2020) reported a positive association between circulation concentrations of IGF-1 and BC risk for premenopausal women, but not for postmenopausal women. In the meta-analysis conducted by Renehan et al. (2004), high concentrations of IGF-1 and IGFBP3 were associated with an increased risk of incident premenopausal BC but not with postmenopausal BC. A pooled data analysis of 4790 cases from 17 prospective studies from 12 countries clearly showed that women with relatively high circulating IGF-1 had a 30% higher risk of BC than women with relatively low circulating IGF-1. This positive association was found in ER+ but not estrogen-receptor negative (ER−) tumors. In addition, this correlation was independent of IGFBP3 and menopausal status (Key et al., 2010). Murphy et al. (2020) in their observational and Mendelian randomization analyses with 430,000 women found evidence that supports a probable causal relationship between circulating IGF-1 concentrations and BC.

Mammographic density is another BC risk factor. With regard to the association between mammographic density and serum IGF-1, there are controversial findings: Diorio et al. (2005) found a positive association in premenopausal women, but other studies did not support this result (Rice et al., 2012; Rinaldi et al., 2014). Recently, Hada et al. (2019) demonstrated a positive association between circulating IGFBP2 and mammographic density particularly among women with lower BMI, but no strong correlation with IGF-1.

Studies investigating the association between the IGF system and BC prognosis are limited and controversial. Some findings suggest a positive correlation (Duggan et al., 2013), others an inverse (Kalledsøe et al., 2019), or no clear association of the biomarkers of the IGF system with all causes of mortality or BC-specific mortality and recurrence (Al-Delaimy et al., 2011; Hartog et al., 2013). Zhu et al. (2020) in their large prospective study showed an inverse and independent association between circulating IGF-1 and all-cause mortality in invasive BC patients, with association being consistent across all clinical risk factors.

## IGF as a Target of Therapy

In hormone-responsive BC cells, IGF-1R function is crucially linked with ER action. In particular, both the IGF-1R and the ER are expressed and act in synergy with estrogen steroid hormone to increase cell proliferation (Radhakrishnan et al., 2011). Otherwise in ER-BC cells, a more aggressive subtype of BC, the levels of the IGF-1R and IRS-1 are often low and IGF is not mitogenic, although IGF-1R is still required for metastatic spread (Radhakrishnan et al., 2011). Additionally, in ER+ cells, estrogens stimulate the expression of the IGF-IR and its major signaling substrate, IR substrate-1 (IRS-1), that promotes estrogen-independence for growth and transformation (Skandalis et al., 2014). Furthermore, IGF-1R and its substrate IRS-1 might induce drug and radio resistance of BC, cells leading to relapse (Pollak, 2012). Besides, high IGF-1R levels in primary tumor samples have been reported to be predictors of shorter disease-free survival, but data on the prognostic value of the IGF-1R for overall survival are contradictory (Pollak, 2012; Yerushalmi et al., 2012). Regarding these evidences, several strategies used to target the IGF axis have been clinically developed for cancer prevention and treatment.

IGF activities are mediated through substrate binding and subsequent activation of IGF-1R (Weroha and Haluska, 2012). The role of the IGF-1R pathway in promoting tumor growth and survival is well established. Targeting the IGF signaling pathway represents a promising approach in the development of novel anti-cancer therapy. The rationale for targeting the IGF-1R is derived widely from cell culture experiments that demonstrate the importance of IGF-IR signaling in promoting proliferation, inhibiting apoptosis, and its involvement and impact on BC cells that are resistant to radiation and chemotherapy (Jones et al., 2009). In BC, specifically the expression of IGF-1R is at least 50% (Ekyalongo and Yee, 2017), much more compared to HER2+ positive BC, which represents 20–25% BC (Wang and Xu, 2019); besides, there is a broader potential group of patients that could be candidates for targeted therapy. In the last few years, different therapeutic strategies have been evaluated to inhibit the IGF-1R signaling pathway. These can be divided into three categories: monoclonal anti-IGF1R antibodies, small molecule tyrosine kinase inhibitors (TKIs), and IGF ligand antibodies. Based on preclinical data, these classes of drug have different profiles of selectivity, efficacy, and toxicity which might have some implications in clinical practice (Burtrum et al., 2003; Maloney et al., 2003). The main clinical trials targeting IGF-1 axis in solid tumors are detailed in Table 1.

**TABLE 1:** Key clinical trial targeting IGF-1 axis in solid tumors.

Title	ID number	Drug regimen	Phase and design	Primary outcome	Status
A dose escalating clinical trial of the IGF-1 receptor inhibitor AXL1717 in patients with advanced cancer	NCT01062620	AXL1717	Ia/b Single arm, open label	RPTD, MTD	Completed, results published
A phase I study of the oral mTOR inhibitor ridaforolimus (RIDA) in combination with the IGF-1R antibody dalotozumab (DALO) in patients (pts) with advanced solid tumors.	NCT00730379	Ridaforolimus plus dalotozumab	I Single arm, open label	Optimal dose, MTD	Completed, results published
A phase 2 study of ridaforolimus (RIDA) and dalotuzumab (DALO) in estrogen receptor positive (ER+) breast cancer	NCT01605396	Ridaforolimus + dalotozumab VS examestane	II Randomized, parallel assignment, open label	PFS	Completed, results published
A phase I trial of the IGF-1R antibody ganitumab (AMG 479) in combination with everolimus (RAD001) and panitumumab in patients with advanced cancer	NCT01061788	AMG 479 + RAD001 VS AMG 479 + RAD001 + panitumumab	I Single center, dose escalation trial	MTD, RPTD	Completed
Phase I study of everolimus (E, RAD001) and ganitumab (GANG 479) in patients (pts) with advanced solid tumors	NCT01122199	Everolimus + ganitumab	I Single arm, open label	MTD, RPTD	Completed
A phase Ib/II study of the combination of BYL719 plus AMG 479 in adult patients with selected solid tumors	NCT01708161	BYL719 (alpelisib) and AMG 479 (ganitumab)	I/II Multicenter, open label, single arm	DLT, ORR	Terminated
The XENERA^TM^ 1 study tests xentuzumab in combination with everolimus and exemestane in women with hormone receptor positive and HER2-negative breast cancer that has spread	NCT03659136	Everolimus + exemestane VS everolimus + exemestane + xentuzumab	II Two arm, open label	PFS	Recruiting
Capecitabine and lapatinib ditosylate with or without cixutumumab in treating patients with previously treated HER2-positive stage IIIB-IV breast cancer	NCT00684983	Capecitabine plus lapatinib ± cixutumumab	II Randomized, parallel assignment, open label	PFS	Completed

*MTD, maximum tolerated dose; RPTD, recommended phase II dose; PFS, progression free survival; DLT, dose limiting toxicities.*

### IGF-1R Antibodies

Monoclonal antibodies that target IGF-1R have shown benefit in early-stage clinical trials (Gualberto and Pollak, 2009). IGF-1 antibodies block ligand binding, inducing receptor internalization and degradation. A few IGF-1R-specific antibodies can also partially affect the IR-A signaling pathway by targeting IGF-1R/IRA hybrid receptors (Wang et al., 2005; Gualberto, 2010). However, they do not inhibit IGF-II activation of IR-A homodimers. One example is MEDI-573 (AstraZeneca), a fully humanized antibody able to neutralize both IGF-I and IR-A pathways *in vitro* and in mice. However, compared to the other human monoclonal antibodies, MEDI-573 selectively inhibits the activation of both the IGF-1R and the IR-A signaling, without cross-reactivity with insulin, sparing the insulin/IR pathway; besides glucose metabolism remains stable (Gao et al., 2011). However, after the completion of phase 2 study in metastatic BC AstraZeneca discontinued the investigation. More recently, another novel IGF ligand neutralizing antibody, Xentuzumab (BI836845) (Boehringer Ingelheim Pharmaceuticals) showed preclinical antitumor efficacy of rapamycin by suppressing IGFs’ bioactivity and inhibiting rapamycin-induced PI3K AKT activation (Adam et al., 2012).

### Receptor Tyrosine Kinase Activity

Another strategy, employed with several agents, is tyrosine kinase inhibition (TKI). This kind of therapy is able to inhibit the kinase domains of the β-subunits of both immunoglobulin and IRs, as their primary sequences share 84% identity in the kinase domains maintaining a relatively intact ATP binding pocket (Munshi et al., 2003). There are only two exceptions to this, NVP-AEW541 and NVD-ADW742. NVP-AEW541 is a small molecular weight pyrrolo-[2,3]-pyrimidine derivative kinase inhibitor of IGF-IR (García-Echeverría et al., 2004), while NVP-ADW742 is an ATP-competitive inhibitor that prevents IGF-IR phosphorylation (Warshamana-Greene et al., 2005). These two inhibitors have 15–30 fold increased potency for IGF-1R kinase inhibition compared to IR kinase inhibition in cellular assay, but they are able to distinguish between the IGF-IR and the closely related InsR (Mitsiades et al., 2004; Serra et al., 2008). TKIs’ lack of selectivity might have some benefit—upregulated serum levels of insulin after IGF-1R monoclonal antibody treatment may not have as much effect on the tumor if both IGF-I1 and IR are blocked. Several studies demonstrated that these TKIs inhibit IGF-IR/IR phosphorylation and AKT activation, and consequently lead to increased apoptosis, decreased *in vitro* cell proliferation, and tumor suppression in xenografts models (Serra et al., 2008; Carboni et al., 2009). However, the potential benefit of TKIs over antibody therapies targeting IGF-I1 might be their capacity to block also IR, which comes at the expense of metabolic alterations such as hyperglycemia and evidence of insulin resistance (Haluska et al., 2006). In 2015 Simon Ekman, in his phase 1a/b study, showed that the oral small molecule IGF-1-receptor pathway modulator had an acceptable safety profile and demonstrated promising efficacy in this heavily pretreated patient cohort, especially in patients with NSCLC (Ekman et al., 2016).

## Crosstalk and Combination Therapies

### Chemotherapy

Cancers have the capacity to develop resistance to traditional therapies, and the increasing prevalence of these drug resistant cancers necessitates wider research and treatment development (Housman et al., 2014). Chemo-resistance is a common problem in the treatment of cancer patients, as cancer cells become resistant to chemical substances used in treatment, limiting the efficiency of chemotherapeutic agents (Phi et al., 2018). When tumor cells are treated with cytotoxic chemotherapy, susceptible cells die, while a subset of resistant cells will continue to proliferate (Weroha and Haluska, 2008). The IGF-pathway is implicated in the chemotherapy resistance process. For instance, IGF-I attenuated the response of theMCF-7 BC cell line to doxorubicin and paclitaxel by at least two mechanisms: induction of proliferation and inhibition of apoptosis (Clemmons, 2007a). Therefore, inhibition of IGF-I action could be useful to cytotoxic chemotherapy in BC. Moreover, it has been evaluated that also the timing of IGF-1R inhibition influences responses to chemotherapy. Zeng et al. showed that the administration of IGF1R inhibitors prior to doxorubicin therapy resulted in the best therapeutic responses registered in BC cell lines. The optimal dosage sequence was doxorubicin followed by an anti-IGF-1R antibody, while the opposite sequence decreased doxorubicin effects (Zeng et al., 2009). Therefore, the timing of IGF-IR inhibition should be considered in the design of future clinical trials, combining IGF-IR blockade and chemotherapy. However, unlike other solid tumors (Goto et al., 2012), in BC, there are no results from clinical trials supporting the hypothesis of whether IGF-1R inhibition will enhance the activity of cytotoxic chemotherapy.

### IGF-1R and Hormonal Therapies

As discussed above, the crosstalk between IGF/IS pathway and estrogen receptors has been widely evaluated for potential new target drugs in ER + BC (Yee and Lee, 2000). ER + BC is the most common subtype, constituting almost 70% of all diagnosed BCs. Therefore, many trials have been performed to verify the efficacy of the combination between anti-IGF-1R and anti-estrogen directed therapies. The overall effect of hormonal agents on the IGF/insulin system is to regulate positively signaling. However, resistance to anti-estrogen therapies is still a pivotal clinical problem (Nahta and Esteva, 2006; Abderrahman and Jordan, 2018). Drug resistance might be partially related to the crosstalk between the ER and the IGF pathways (Fagan et al., 2002). For instance, HBL100 cells, under tamoxifen therapy, are not able to proliferate, but if they are treated concomitantly with IGF, they could survive (Christopoulos et al., 2018). However, the majority of clinical trials evaluating the combination of anti-IG-1R and anti-ER therapies in endocrine-resistance BC have yielded disappointing results, as they did not lead to any improvement in clinical outcome (Kaufman et al., 2010). Most of the women enrolled in these trials had already developed resistance and anti-IG-1R strategies were tested as the second and third line of therapy. The lack of clinical success of these trials implies that targeting just IGF-1R is not enough to overcome tumor growth. It has been reported that the continuous exposure of MCF-7 cells to tamoxifen resulted in the eventual emergence of resistant cells, called MCF-7 Tam-R, which lose IGF-IR expression but maintain IR expression for their growth (Fagan et al., 2002). Considering these results, targeting the IR pathway could be an alternative option to treat TamR BC.

### IGF-1R and PI3K/Akt/mTOR Axis

Insulin-like growth factor-1 signaling is involved in complex cross-talk with other receptor tyrosine kinases (RTLs) and their downstream effector which could likely confer resistance to inhibitors of a single class of receptor (Wilson et al., 2012). As known, the PI3K/AKT and RAS-MAPK axes are two well-established downstream pathways of IGF/insulin signaling. It is understood that the AKT pathway could be reactivated despite IGF-1R downregulation, mediated by anti-IGF-1R antibody or TKIs, leading to tumor progression (Cao et al., 2008). Based on this evidence, PI3K inhibitor such as LY294002 (Clark et al., 2002), S6K1 inhibitor H89 (Becker et al., 2011), MAPK inhibitor U0126 (Becker et al., 2011; Casa et al., 2012), and dual PI3K/mTOR inhibitor NVP-BEZ235 (Brachmann et al., 2009) have been studied in pre-clinical and clinical studies supported by the hypothesis that combinations of AKT and IGF-IR/InsR inhibitors would be an effective treatment against hormone-independent ER + BC (Fox et al., 2013). Several studies have also shown that the dual inhibition of IGF-IR and m-TOR increased antitumor activity both *in vitro* and in BC. Di Cosimo et al. (2010), in a phase 1 clinical trial, have demonstrated clinical benefit in 21.7% of BC patients combining ridaforolimus (a small molecule inhibitor of mTOR) and IGF-1R antibody dalotuzumab. The combination was feasible and well tolerated and a phase 2 was initiated, but accrual was prematurely interrupted due to a higher than expected incidence of stomatitis in the treated patients (Rugo et al., 2017).

Vlahovic et al. have evaluated the clinical benefits of combining ganitumab, a monoclonal antibody directed versus IGF-1R, with everolimus (Ev) and panitumumab in patients with advanced cancers. However, the triplet regimen of ganitumab, Ev, and panitumumab was associated with unacceptable toxicity, and clinical activity has been demonstrated only in NSCLC and sarcoma (Vlahovic et al., 2018). Moreover, another phase I study of Ev and ganitumab in patients with advanced solid tumors has shown that this combination is safe; nevertheless, prolonged clinical benefit [stable disease (SD) ≥ 20 weeks] was noted only in refractory fibrolamellar HCC, neuroendocrine, GIST, and urachal cancers (Jalal et al., 2013). A phase Ib/II study (NCT01708161) investigated the maximum tolerated dose (MTD) and response rate of the combination of ganitumab with alpelisib, a small molecule inhibiting the subalpha of PI3-kinase, in patients with ovarian and hormone receptor positive cancer carrying the somatic PIK3CA mutation. However, the recruitment has been stopped due to inconclusive results.

Recently, a new IGF-1 monoclonal antibody, Xentuzumab (Xen) has been investigated in the phase II XENERA-1 trial in combination with Ev and exemestane (Ex) in post-menopausal women with ER+ and HER2− metastatic BC (Crown et al., 2019). Crown et al., at San Antonio Breast Cancer Symposium in 2018, showed that in the overall (randomized) population, progression-free survival (PFS) was not significantly improved in patients treated with Xen + Ev + Ex compared with Ev + Ex (Schmid et al., 2019). However, a pre-specified subgroup analysis showed that in the non-visceral metastases subgroup, the Xen + Ev + Ex arm demonstrated favorable PFS compared with the Ev + Ex arm. Specifically, an ongoing Phase II study (NCT03659136) is investigating the use of Xen + Ev + Ex in post-menopausal women with HR+/HER2− LA/mBC and non-visceral disease (Nahta et al., 2005).

### IGF-IR and HER2/erbB Receptor Therapy

Most of the patients who obtain an initial response to trastuzumab-based therapy develop resistance within 1 year after commencing treatment (Albanell and Baselga, 2001). The possible existence of bi-directional crosstalk between the erbB family of receptors and IGF-1R may be implicated in resistance to targeted therapies including these receptors pathways (Weroha and Haluska, 2008). In BC cell models that overexpress HER2, an increased level of IGF-IR signaling might interfere with the action of trastuzumab (Lu et al., 2001). Moreover, BC cell lines, cultured in combination with an anti IGF-1 antibody, showed an increased cytotoxic effect when treated with trastuzumab (Albanell and Baselga, 2001). Thus, strategies that co-target HER-2 and IGF-1R may prevent or postpone development of resistance to trastuzumab (Browne et al., 2012). In BC, an IgG1 monoclonal antibody that binds IGF-1R, cixutumumab (IMC-A12) is being investigated in combination with lapatinib in a phase II trial (Haluska et al., 2014). The mechanisms related to IGF-1R-driven HER-2 therapy are not well known; nevertheless, some studies showed that HER-2 therapy resistance may be associated with the downregulation of the PTEN/PI3K/AKT signaling pathway (Gallardo et al., 2012). Despite this, it has been shown that HER-2 overexpressing cancers treated with PI3K inhibitors developed AKT-mediated activation of other tyrosine kinase growth factors such as IGF1-R, Ins-R, and HER3 treatment. Besides, PI3K inhibitors should be combined with HER-2 targeted therapies including trastuzumab or lapatinib, in order to avoid AKT signaling activation (Chakrabarty et al., 2012). Moreover, in trastuzumab-resistant tumors, IGF-1R cell motility is related to the stimulation of FAK signaling and Forkhead box protein M1 (FoxM1). Furthermore, trastuzumab-resistant cancer cells might be the best candidates for anti-HER2 and anti-IGF-1R combined therapies (Sanabria-Figueroa et al., 2015).

## Future Directions

Novel approaches to target IGF/insulin systems are related to small interfering RNA (siRNA) and microRNA (miRNA) in order to reduce IGF-IR expression and function (Jung and Suh, 2012). Durfort et al. showed that silencing IGF-IR using synthetic siRNA bearing 29-O-methyl nucleotides could induce cell-cycle arrest and decrease cell proliferation. Moreover, this study suggested that the crosstalk between the IGF-I axis and antitumor immune responses can mobilize pro-inflammatory cytokines, offering a new clinical approach for treatment of mammary tumors expressing IGF-IR (Durfort et al., 2012). Nevertheless, this approach has two main problems: the first one is that siRNA formulations for systemic application face a series of hurdles *in vivo* before reaching the cytoplasm of the target cell (Whitehead et al., 2009) and the second is the transient inhibition of the IGF pathway. However, preclinical *in vivo* studies showed that it might be possible to overcome at least the second obstacle, with the development of stable *in vivo* and inducible long-term expression of target short hairpin RNA using dimerizing drugs such as doxycycline or tetracycline (Jones et al., 2009). Furthermore, other miRNAs were investigated in the past few years (Guo et al., 2013). For instance, decreased levels of miR-139, which targets IGF-IR in colorectal cancer (CRC), were associated with disease progression and metastasis. This re-expression of miR-139 might suppress CRC cell invasion and metastasis by targeting IGF-IR (Shen et al., 2012). In esophageal squamous cell carcinoma (ESCC), it has been shown that miR-375 inhibits tumor growth and metastasis through repressing IGF-1 receptor (Kong et al., 2012). Maybe in the future, siRNAs targeting IGF-IR will be modified in order to improve the effect of IGF-IR downregulation and consequently modulate antitumor immune responses with the aim to offer a new clinical approach for treatment of mammary tumors expressing IGF-IR.

## Conclusion

The IGF system has been involved in the oncogenesis of the majority of solid tumors. The central implications of this pathway in tumor cell proliferation and metastasis makes it an important therapeutic target. In BC, the IGF pathway has been implicated in resistance to the three cornerstones of BC therapy: hormonal agents, HER receptor targeting agents, and cytotoxic chemotherapy. Therefore, several clinical trials are currently evaluating the efficacy of IGF-1R inhibition to overcome these resistance mechanisms. The competitive landscape for anticancer therapies in BC and the difficulty to recruit a sufficient number of patients limited *de facto* the continuation and validation of research with IGF-1R and GF inhibitors. That is why, even considering the encouraging initial results that we have illustrated, combined with the enormous potential clinical impact of the IGF axis, there is not yet an optimal combination therapy paradigm.

## Author Contributions

AI, MS, and OB drafted the manuscript. DG conceptualized the study and revised the manuscript. All authors contributed to the article and approved the submitted version.

## Conflict of Interest

The authors declare that the research was conducted in the absence of any commercial or financial relationships that could be construed as a potential conflict of interest.
